# Supplementary material for: Exploring perspectives on digital smoking cessation just-in-time adaptive interventions: A focus group study with adult smokers and smoking cessation professionals
Source: PLOS Digit Health. 2025 May 2;4(5):e0000705. doi: 10.1371/journal.pdig.0000705 (PMC12047773; doi:10.1371/journal.pdig.0000705)
Supplement: S4 File — (DOCX) [file pdig.0000705.s004.docx]

Screening Survey Smokers

# A: Baseline screening questionnaire

| 1. **What is your age (in years)?** | Free text (validated as number) |
| --- | --- |
| 1. **Do you smoke cigarettes at all nowadays?** | 1. No 2. Yes |
| 1. **Do you smoke cigarettes every day?** | 1. No 2. Yes |
| 1. If Yes to 3): **How many cigarettes do you smoke per day on average?** | Free text (validated as number) |
| 1. If Yes to 2): **Which of the following best describes you?** | 1. I don’t want to stop smoking 2. I think I should stop smoking but don’t really want to 3. I want to stop smoking but haven’t thought about when 4. I REALLY want to stop smoking but I don’t know when I will 5. I want to stop smoking and hope to soon 6. I REALLY want to stop smoking and intend to in the next 3 months 7. I REALLY want to stop smoking and intend to in the next month |
| 1. **Do you have a smartphone that you use daily?** | 1. No 2. Yes |
| 1. **Do you live in London?** | 1. No 2. Yes |
| 1. **Do you live in the UK?** | 1. No 2. Yes |
| 1. If Yes to 7): **Are you willing to attend a focus group session either** **in-person at UCL (Bloomsbury, Central London) or remotely online via MS Teams?** | 1. No 2. Yes, both in-person at UCL (Bloomsbury, Central London) or remotely online via Zoom 3. Yes, in-person at UCL (Bloomsbury, Central London) 4. Yes, remotely online via Zoom |
| 1. If No to 7): **Are you willing to attend a focus group session remotely online via MS Teams?** | 1. No 2. Yes |

# B: Additional baseline questions for eligible participants

| 1. **What is your name?** | Free text |
| --- | --- |
| 1. **What is your email address? (so we can contact you to arrange a time for the focus group)** | Free text (validated as email) |
| 1. **What is your mobile phone number? (so we can contact you to arrange a time for the focus group)** | Free text (validated as phone number) |
| 1. **Which of the following genders do you identify with?** | 1. Male 2. Female 3. In another way 4. Prefer not to say |
| 1. **Which of the following ethnicities do you identify with?** | **Asian or Asian British**   1. Indian 2. Pakistani 3. Bangladeshi 4. Chinese 5. Any other Asian background, please specify:   **Black, Black British, Caribbean, or African**   1. Caribbean 2. African 3. Any other Black, Black British, or Caribbean background, please specify:   **Mixed or multiple ethnic groups**   1. White and Black Caribbean 2. White and Black African 3. White and Asian 4. Any other Mixed or multiple ethnic background, please specify:   **White**   1. English, Welsh, Scottish, Northern Irish, or British 2. Irish 3. Gypsy or Irish Traveller 4. Roma 5. Any other White background, please specify:   **Other ethnic group**   1. Arab 2. Any other ethnic group, please specify: 3. Prefer not to say |
| 1. **Do you have caring responsibilities? (select all that apply)** | 1. None 2. Parent, guardian, or carer of a child/children (under 18) 3. Carer of a disabled adult (18 or over) 4. Carer of an older person 5. Prefer not to say |
| 1. **What type of job do you do?** | 1. Routine or manual (e.g., plumber, bricklayer, painter, driver, hairstylist, caretaker, shop assistant, waiter) 2. Non-manual (e.g., bank clerk, teacher, nurse, lawyer, accountant, librarian, engineer, programmer) 3. Full-time student 4. Unemployed 5. Other (e.g., retired) |
| 1. **Do you have any post-16 qualifications (e.g. T-levels, A-levels, level 3 NVQ, Scottish Advanced Highers, university degree)?** | 1. No 2. Yes |
| 1. **How soon after waking do you have your first cigarette?** | 1. Within 5 minutes 2. 6-30 minutes 3. 31-60 minutes 4. More than 60 minutes |
| 1. **What percentage of the cigarettes you smoke are handrolled (as opposed to factory-made)?** | Slider to allow selection of any number 0 to 100 |
| 1. **At what age did you start smoking regularly?** | Free text (validated as number & not higher than current age) |
| 1. **Have you ever made a serious attempt to quit smoking? By serious we mean you decided that you would try to make sure you never smoked again.** | 1. No 2. Yes, but not in the past year. 3. Yes, in the past year. |
| 1. If yes to 12): **How many serious attempts to quit smoking have you made in your life?** | Free text (validated as number) |
| 1. **Have you ever used any of the following to help you stop smoking or cut down the amount you were smoking? (select all that apply)** | 1. Nicotine replacement product (e.g., patches/gum/inhaler) without a prescription 2. Nicotine replacement product on prescription given to you by a healthcare professional 3. Zyban (bupropion) 4. Champix (varenicline) 5. Cytisine 6. E-cigarette or another vaping device 7. Heated tobacco product (e.g. iQOS with HEETS, heatsticks) 8. Tobacco-free nicotine pouch/pod or 'white pouches' that you place on your gum (e.g., Zyn, On!, Nordic Spirit, Velo, Lyft, Skruf) 9. Attended a Stop Smoking group 10. Attended one or more Stop Smoking one-to-one counselling/ advice/ support sessions 11. Acupuncture 12. Hypnotherapy 13. Phoned a smoking helpline 14. A book or pamphlet 15. Visited a website 16. Used an application (app) on a computer, tablet, or smartphone 17. Support from an online community 18. A text messaging service 19. None of these 20. Other, please specify: |
| 1. **Have you ever tried to change or monitor a health behaviour with the help of digital technology? This could include tracking your steps using a pedometer, using an app to help you monitor your food or alcohol intake, or using an app or website to guide your workouts** | 1. No 2. Yes, please specify (name, platform, behaviour targeted, how often/for how long you have used it): (short text box, no more than 70 words) |
| 1. If selected 2) or 3) at A9): **What are you preferred dates to come into UCL (Bloomsbury, Central London) for a one-off focus group? (select all that apply)** | 1. TBD 2. TBD 3. TBD 4. TBD 5. TBD |
| 1. If selected 2) or 4) at A9)/selected 2) to A10): **What are you preferred dates to attend a one-off focus group remotely via MS Teams? (select all that apply)** | 1. TBD 2. TBD 3. TBD 4. TBD 5. TBD |
| 1. **Where did you hear about this study?** | 1. Physical poster 2. Facebook 3. Instagram 4. Friend recommendation 5. Other |
| 1. **Would you like to receive a plain language summary of the study results when they are ready?** | 1. No 2. Yes |
