# Supplementary material for: Exploring perspectives on digital smoking cessation just-in-time adaptive interventions: A focus group study with adult smokers and smoking cessation professionals
Source: PLOS Digit Health. 2025 May 2;4(5):e0000705. doi: 10.1371/journal.pdig.0000705 (PMC12047773; doi:10.1371/journal.pdig.0000705)
Supplement: Attachment — Submitted filename: Response to Reviewers 1.1.docx [file pdig.0000705.s010.docx]

# Response to Reviewers

### Reviewer #1

Reviewer #1: I have reviewed the article: PDIG-D-24-0522 ‘Exploring perspectives on digital smoking cessation just-in-time adaptive interventions: A focus group study with adult smokers and smoking cessation professionals’
This is an interesting report and an important area of research. Nevertheless, I have a few comments that I think may improve the manuscript.
*We thank the reviewer for their time in reviewing our paper and providing really helpful suggestions which we have incorporated as described below.*

On page 9, line 199 you state that the topic guides were informally piloted. It would be useful to provide additional detail about the pilot structure in the text and if any changes were made to the topic guide as a result.
*We have provided some additional detail on the piloting process on p10: “In addition, the finalised topic guides underwent informal piloting through a mock focus group. This group comprised non-researchers known to the authors who had some experience with smoking but were largely unfamiliar with the research area. Insights from this process led to minor refinements in the wording of certain questions and adjustments to the presentation slides to improve clarity.”*

Participants in general reported both a low level of smoking and low motivation to quit- this is a limitation as the insights of this group may be quite different from a group that contains heavy smokers or those with a greater motivation to quit. I would recommend including this as a limitation in the discussion.
*We have added this as a limitation on p38: “A further limitation is that participants generally reported lower levels of smoking and lower levels of motivation to quit. While this partly reflects the changing characteristics of smokers in England (85,86), it also means our findings may not generalise to people who smoke at higher levels or who have a higher motivation to quit.”*

I understand why smoking cessation professionals’ data and that of the smokers were analysed together, however as they are such different groups with different motivations and experiences, at times I find that the two don’t necessarily fit together all that clearly. However I appreciate it may be impractical to have two separate analyses and will leave that to the research team to decide.
*We appreciate the reviewer’s concern about combining data from smoking cessation professionals and individuals who smoke. While these groups have different perspectives, we believe that their insights complement each other in ways that strengthen the analysis, as similar themes emerged in the analysis of these two separate stakeholder groups. Framework Analysis was also chosen as the analysis method because it allowed us to draw out these complementary insights. We also believe that presenting results from both stakeholder groups together provides a more well-rounded understanding of how a JITAI should be designed and implemented to meet stakeholder and user needs, expectations, and preferences. We have added the following rationale to the introduction (pp5-6): “This study involves two groups of stakeholders, namely smokers and smoking cessation professionals, due to the complementary insights they can offer on how JITAI could or should be implemented (30). Smokers can provide insight into the lived experience of quitting and use of support, personal motivators, practices, and preferences, while smoking cessation professionals can provide insights into feasibility and adherence, common patterns, and integration with healthcare services.” We have also added the following to address this issue in the limitations (p38): “Another potential limitation is combining data from smoking cessation professionals and smokers, who have different motivations and experiences. However, similar and complementary themes emerged in analysing the data from these two separate stakeholder groups, allowing a more well-rounded picture of stakeholder and user needs, expectations, and preferences for JITAI design and implementation to emerge.”*

### Reviewer #2

Reviewer #2: I have thoroughly reviewed the manuscript. In my assessment, the work does not currently demonstrate a high degree of innovation. However, I have included detailed recommendations aimed at enhancing the manuscript's overall contribution. I hope these suggestions prove helpful.
*We appreciate the reviewer’s time and thoughtful feedback, which we believe has helped improve it. Please find point-by-point responses below.*

Abstract

1.abstract mentions the Theoretical Domains Framework (TDF) and Technology Acceptance Model (TAM). Expand slightly on how these frameworks guided the analysis. Were they primarily used for deductive coding? Give a one-sentence indication of the key constructs from these frameworks that emerged as relevant.
*We have expanded the abstract to clarify that TDF and TAM were primarily used for deductive coding: “Transcripts were analysed using inductive and deductive Framework Analysis; deductive codes were derived from the Theoretical Domains Framework and the Technology Acceptance Model.” However, due to the abstract world limit, we were not able to specifically highlight key constructs from these frameworks in the abstract. Themes and codes that emerged from these deductive frameworks are, however, highlighted in the results and in the supplementary material as deductive codes.*

2.Saying "four major themes... were identified" isn't as impactful as it could be. Indicate, even roughly, the prevalence or relative importance of these themes. Did one theme emerge far more strongly than others?
*None of the themes emerged more strongly than the others; instead, they were interlinked and informed each other as co-equal pillars of the analysis. We briefly mention this in the abstract now (“Four co-equal major themes, “Smoking Cessation Process”, “JITAI Characteristics”, “Perceived Value of the JITAI”, and “Relationship with the JITAI”, and 16 subordinate themes were identified.”), and restate this with slightly more detail at the beginning of the results section on p18: “Although the theme “JITAI Characteristics was most frequently discussed, none of the themes was dominant; rather, the themes interlink and inform each other.”*

3.current concluding sentence ("JITAIs will need to balance aspects...") is rather generic. Sharpen the specific implications of your findings for JITAI design. What is the novel takeaway regarding this balance?
*We tried to highlight the implications a bit more clearly, as far as possible given the word count: “JITAIs balance aspects of competing demands in their design, such as optimising for both convenience and sufficient disruption, promoting autonomy, and integrating interruptive and ambient content while also meeting stakeholder needs and expectations in terms of privacy.”*

4.author summary uses the pronoun "we" without defining who "we" are.
*We reworded the authors summary to no longer include first-person pronouns.*

Introduction

1.While the introduction establishes the importance of smoking cessation and the potential of JITAIs, more could be done to highlight the specific gaps in the current JITAI literature that this study addresses. What are the limitations of existing JITAIs that this research seeks to overcome?
*We have clarified and expanded on the research gap in the introduction on p5: “Additionally, there is little research on stakeholder needs, expectations, and preferences for smoking cessation JITAIs specifically. Since JITAIs function differently from other digital interventions and the ever-changing landscape of digital interventions, previous work on other types of digital interventions may be of limited applicability.”*

2.rationale for including both smokers and smoking cessation professionals is good, but it could be further strengthened. Expand on the unique insights each group brings to the table. What specific knowledge or experience does each group possess that the other lacks?
*We tried to strengthen this rationale by adding the following two sentences on pp5-6: “This study involves two groups of stakeholders, namely smokers and smoking cessation professionals, due to the complementary insights they can offer on how JITAI could or should be implemented (30). Smokers can provide insight into the lived experience of quitting and use of support, personal motivators, practices, and preferences, while smoking cessation professionals can provide insights into feasibility and adherence, common patterns, and integration with healthcare services.” We have also added the following to address this issue in the limitations (p38): “Another potential limitation is combining data from smoking cessation professionals and smokers, who have different motivations and experiences. However, similar and complementary themes emerged in analysing the data from these two separate stakeholder groups, allowing a more well-rounded picture of stakeholder and user needs, expectations, and preferences for JITAI design and implementation to emerge.”*

3.Make the connection between the research questions and the practical considerations of JITAI design more explicit. How will answering these questions directly inform decisions about intervention features, content, and delivery mechanisms?
*We added the following sentence to make the link between the research questions and the practical design considerations more explicit on p6: “By addressing these questions, we aim to inform JITAI design by identifying preferred features (e.g., real-time support vs. ambient content) and more general design considerations and principles to meet stakeholder needs, expectations, concerns, and preferences.”*

4.While the study recognizes the value of autonomy and personalization, it could expand on how these features can be used to address health inequities among diverse populations.
*Since autonomy and personalisation were identified as important themes from the data rather than major a priori concerns, we expanded on this in the discussion section on p33: “Previous research highlights that transparency and agency might be particularly important for underserved and marginalised groups (25), meaning that incorporating these principles may be an avenue to promote health equity. Furthermore, our analyses suggest that potential users highly value personalisation and the ability to flexibly fit an intervention into one’s life, which JITAIs can enable to a greater extent than previous digital smoking cessation interventions. This aligns with the emphasis put on personalisation in previous literature and recommendations (51–53). In terms of personalisation, previous research indicates that users appreciate interventions that are tailored to their values or culture (55) and that, based on their personality, people are differentially responsive to different persuasion strategies (56). Tentative evidence also suggests that tailoring and targeting may make interventions more effective (57,58). Enabling autonomy and personalisation may also help address health inequalities by allowing users to tailor a JITAI to their specific preferences and circumstances. The ability to customise the content or modality or to restrict data-sharing can reduce barriers to engagement, particularly for underserved populations who may face accessibility challenges or distrust in health services (25,59,60).”*

Methods

1.manuscript mentions a pragmatist epistemology, but doesn't explain what that means in the context of this study. How did this epistemological stance influence the research design, data collection, or analysis?
*We expanded on this and now explicitly link the epistemology to the research design on p7: “This study was designed as part of a mixed-methods project with a pragmatist epistemology (32,33). This means that the project and this study are primarily concerned with gaining relevant, actionable knowledge from various sources that describe the problem at hand in its context but may not necessarily translate into definite scientific truths (32,33). Therefore, this study asked questions and analysed data to directly address considerations relating to the complex, real-world intervention context and involved two groups of stakeholders with complementary perspectives.”*

2.While the rationale for the number of focus groups and participants is provided, strengthen the argument for data saturation. What specific criteria were used to determine when saturation was reached?
*We have provided some further information on saturation by adding the following sentences on p8: “While saturation was not formally assessed, all but one minor theme were discussed across multiple groups and no one single group substantially changed the meaning of any themes. This indicates theoretical and meaning saturation, in line with commonly used definitions (35).”*

3.While the topic guides are included as supporting information, provide more detail within the main text about the key areas explored in each guide. What specific prompts or probes were used to elicit participant perspectives on JITAI features, content, and integration with healthcare systems?
*We have added some additional details including example prompts to the main text on p10: “Topic guides addressed the integration of a JITAI into its users’ lives and care provision, preferred content and features, and data and privacy. Questions and prompts included “How do you feel about the idea of the JITAI collecting, storing, and analysing data about your smoking habits and cravings?” (smokers) and “How could a JITAI be integrated into the services and support you provide?” (smoking cessation professionals).”*

4.Expand on the process of developing the codebook. What specific steps were taken to ensure inter-coder reliability and consistency in coding?
*In this study, coding was conducted by a single researcher, meaning that assessing inter-coder reliability was not applicable. However, to ensure rigour and consistency in our coding process, we adopted a systematic approach to developing and refining the codebook, as detailed on p11: “CL led the analysis and created the initial codes and themes. The codes, themes, and their relation to one another were then discussed and agreed upon by the whole research team. A codebook describing the themes and their definitions was iteratively developed in discussion with the whole research team. This collaborative process ensured that the coding framework was coherent, clearly defined, and consistently applied, thereby enhancing the reliability of the analysis mainly conducted by one coder.”*

Results

• While the results section presents demographic data on participants, integrate this information more effectively into the qualitative findings. How did participant characteristics relate to their perspectives on JITAIs?
*Where participants explicitly drew connections between characteristics, such as age, and their views, we have now presented this in the results section (“For example, participants who were parents mentioned integrating and acknowledging that their child was an important motivator for them to quit smoking would be helpful for them, but inapplicable for people who do not have children.”, p23). In the data collection or analysis, we did not detect any specific latent patterns or distinctions in terms of how participant characteristics related to their specific perspectives in JITAI, aside from the differences between smokers and smoking cessation professionals that we have already pointed out.*

• While the results section includes quotations, increase the frequency and diversity of participant voices. Select quotations that are particularly compelling, insightful, or representative of different perspectives.
*In line with common practice and recommendations for qualitative research, we aimed to keep to one or two quotations per theme. Quotations were selected based on authenticity and how well they encapsulate or illustrate the meaning of a specific theme, in line with recommendations for the use of quotations to illustrate findings (Lingard, 2019. DOI: 10.1007/s40037-019-00550-7; Eldh, Årestedt, & Berterö, 2020. DOI: 10.1177/1609406920969268). We have now stated this explicitly in the analysis section of the paper on p11: “Quotations are provided to illustrate findings and more richly describe themes (49).”*

*However, we appreciate the suggestion and have included the additional quotations (see below), focussed on including more diversity of voices.*

“I think it would depend on how long I had the cravings for and how useful the app is. If it’s enjoyable to use and I’ve found it helpful then it would last longer” (Smokers Group 2, 27M, non-manual occupation) (p 19) (Theme: Time and Stability)

“I think sometimes it gets in your head and we… we make it toxic for one another.” (Smokers Group 1, 23F, routine or manual occupation) (p 21) (Theme: Social Influences)

“I suppose there’d be like a number of approaches in the app. There would be notifications and data, and this, that, and various things. So, which some people might have no interest or in one of them, or, or another. Perhaps at the beginning of your quitting journey you sort of predefine what methods you are going to be using and that might not be all of the methods within the app, so turn them off then.” (Smokers Group 3, 22F, full-time student) (p24) (Theme: Flexibility and Personalisation)

“It’s giving you that sense of partnership, but at the same time not being overly invasive.” (Smokers Group 1, 43M, non-manual occupation) (p29) (Theme: Relationship with the JITAI)

“[F]or me, it’s really important that I get to make certain decisions first.” (Smokers Group 1, 23F, routine or manual occupation) (p30) (Theme: Autonomy and Choice)

“For real I would feel more comfortable if I knew exactly what data is being collected and how it’s being used. I think there should be clear guidelines around data privacy and security, and people should have the option to delete their data if they wish.” (Smokers Group 1, 20M, full-time student) (p31) (Theme: Privacy and Safety)

•manuscript mentions that perspectives on user engagement and privacy diverged between stakeholder groups. Quantify the extent of these differences and provide more detailed examples of these contrasting viewpoints.
*Given the design and epistemology of this study, we do not believe that quantifying these differences is appropriate (cf. Monrouxe and Rees, 2019. DOI: 10.1111/medu.14010; Neale, Miller, & West, 2014. DOI: 10.1111/add.12408). However, we have clarified that professionals’ emphasis on the importance of privacy was unanimous (p30): “Smoking cessation professionals were unanimously very concerned about privacy and safety, and while most smokers also highly valued privacy, they expressed more ambivalence about its importance.”*

•demographic data is described but does not consider how characteristics such as "educational attainment" affect views on JITAIs.
*As mentioned above, we did not detect any specific latent patterns or distinctions in terms of how participant characteristics related to their specific perspectives in JITAI and the study was not designed to look at differences based on these characteristics. We do consider the differences between smokers and smoking cessation professionals: “Smoking cessation professionals and smokers raised largely similar themes, although some of the specific perspectives, particularly regarding user engagement and privacy and data protection diverged between stakeholder groups.” (p18); “Additionally, some participants suggested integrating a JITAI into a multidisciplinary or lifestyle intervention to increase longevity and use, while others, primarily smoking cessation professionals, expressed concerns about overloading users and keeping the intervention focused. However, smoking cessation professionals also mentioned that some sort of integration with carers or primary care providers may be beneficial as an accessibility adaptation for users with disabilities or older users.” (p25); “Both smokers and smoking cessation professionals expressed concerns about the potential burden of completing EMAs. They agreed that filling out more than a few per day would be unrealistic, with smoking cessation professionals being particularly sceptical about users completing more than two.” (p27); “There was some dissent in this theme. Smoking cessation professionals were unanimously very concerned about privacy and safety, and while most smokers also highly valued privacy, they expressed more ambivalence about its importance.” (p30)*
*We have now also added occupational status as a descriptor for quotations of smokers.*

Discussion

1.discussion compares the findings to existing literature, but it could be more nuanced and critical. What are the key points of convergence and divergence between this study's findings and previous research? Are there any findings that contradict or challenge existing assumptions?
*To make the comparison to the existing literature more nuanced and critical, we have added the following paragraph (pp32-33): “The emphasis on the importance of personalisation, engagement strategies, and user autonomy is in line with previous research (52–59). However, we also identify novel considerations, such as the inherent tension of balancing convenience with sufficient disruption to drive behaviour change, which has not been discussed in prior research. Additionally, while most prior research consistently highlights privacy as a major concern (54,60), our findings suggest more varied user perspectives, with some smokers exhibiting privacy cynicism and a willingness to trade data for perceived benefits.”*

2.discussion identifies a tension between convenience and disruption, but it doesn't fully explore the implications of this finding. How can JITAIs effectively balance these competing demands? What specific design strategies can be used to achieve this balance?
*We added the following sentences to outline potential strategies to balance these competing demands (p35): “Balancing these competing demands in the design and implementation of JITAIs may be difficult. One potential solution is to implement user-controlled engagement settings, allowing for the customisation of the maximum or minimum notification frequency or the content delivery modes. Additionally, adaptive algorithms that adjust intervention delivery based on user response patterns could help maintain engagement. Finally, opt-in reinforcement strategies, such as gamification and milestone-based rewards, could be used as tools to influence the convenience-burden trade-off calculus.”*

3.limitations section could be more specific about the potential impact of the study's limitations on the generalizability of the findings. How might the findings differ if the study had included a more diverse sample?
*We have expanded the limitations section to more clearly state how the sample characteristics limit the generalisability of the findings (pp37-39): “Additionally, our sample was UK-based and skewed towards a young and highly educated demographic, although it was ethnically diverse. As a result, our sample may not have fully captured the barriers to JITAI use that older adults or individuals with lower educational attainment might face. This skew may limit generalisability as there is an existing digital divide, i.e. socioeconomic differences in access and barriers to the use of digital (health) interventions (81). Previous research indicates that digital health interventions are disproportionately used by people in positions of social advantage (67,82), although some evidence also suggests that this relationship may be more complex than a straightforward linear association (83). Therefore, even digital health interventions that are overall beneficial to population health may widen health inequalities (84), particularly when developed without meaningful stakeholder involvement. However, some previous research also suggests that digital smoking cessation interventions have the potential to be more effective for individuals in disadvantaged socioeconomic positions than for those in more advantaged groups (67,85). Our sample was also limited to people with reliable internet access or the ability to commute to central London, who spoke English fluently, and who did not require any assistive technology to participate. While smoking cessation professionals raised issues related to access for populations who would not be able to participate in a focus group scenario such as this, not hearing from people directly affected means that crucial concerns relevant to them might have been missed. A further limitation is that participants generally reported lower levels of smoking and lower levels of motivation to quit. While this partly reflects the changing characteristics of smokers in England (86,87), it also means our findings may not generalise to people who smoke at higher levels or who have a higher motivation to quit. Another potential limitation is combining data from smoking cessation professionals and smokers, who have different motivations and experiences. However, similar and complementary themes emerged in analysing the data from these two separate stakeholder groups, allowing a more well-rounded picture of stakeholder and user needs, expectations, and preferences for JITAI design and implementation to emerge. Additionally, multiple participants mentioned attaching a potential JITAI to the NHS, an institution that has a unique role within British society and cultural, political, and social discourse (88,89). Therefore, it is reasonable to assume that certain preferences, especially those concerning the affiliation and embedding of a JITAI, will not be generalisable beyond the British context. Another important consideration is the rapid evolution of technologies such as JITAIs. As these technologies develop, improve, and become more widespread, it is likely that stakeholders’ opinions on them will change (90). Therefore, the longevity of our findings is uncertain.”*

4."research team and reflexivity" section can be mentioned in the discussion to explain how the researchers' academic backgrounds may influence the way data was collected and interpreted.
*We explain the influence of researcher characteristics on data collection and interpretation in the methods section (p12): “Therefore, the research team’s experience with smoking and smoking cessation is primarily academic rather than based on individual lived experience, and they have a general belief that JITAIs and digital smoking cessation interventions more broadly have the potential to improve public health and are worth exploring. Although the team tried to be led by participants and the data in their analyses, this will likely have informed their view when analysing the data.
Study participants did not know the focus group facilitators or the wider research team and had little contact before the focus groups except for a reminder email or video call. The research goals were briefly reiterated to the participants at the start of each focus group session. However, care was taken not to overemphasise them to reduce potential social desirability effects.”
We have also added the following sentence to the limitations section (p39): “Furthermore, the fact that qualitative research is an inherently interpretive exercise means that, as mentioned above, although the team tried to be led by participants and the data in their analyses, this will likely have informed their view when analysing the data.”*

5.conclusion should provide a clear and compelling call to action for researchers and practitioners. What specific steps should they take to translate the findings of this study into practice? *We have added an implication for practice section to provide clearer, actionable recommendations for practitioners and developers (p39): “The findings of this study suggest that JITAI developers could prioritise personalisation and flexibility to create engaging, companion-like interventions. These elements, along with transparent data policies, could also support stakeholder priorities such as autonomy and privacy. Additionally, developers should carefully balance convenience with sufficient disruption to drive behaviour change, tailoring this balance to the specific intervention and target population. While social support features and healthcare integration may be of value, the findings of this study suggest these should remain optional rather than essential components.”*

6.conclusion should clearly articulate the unique contribution of this study to the existing literature. What new knowledge or insights does this study offer that were not previously available?
*We have rephrased the conclusion to better highlight the unique contribution of this study to the existing literature (p40): “This study is one of the first studies to qualitatively explore different stakeholders’ perspectives on JITAIs for smoking cessation. While some of the findings align with existing research on other types of digital interventions, such as the importance of personalisation, features to promote engagement, and user autonomy, the study also highlights some previously unexplored considerations, such as a tension between convenience and sufficient disruption to support behaviour change. Additionally, the findings indicate that stakeholder perspectives on privacy are complicated: while privacy is highly valued by smoking cessation professionals and some smokers, some smokers also display privacy cynicism and a willingness to trade privacy for perceived effectiveness or ease of use.”*

7.conclusion should consider the long-term implications of this research for the field of smoking cessation. How might JITAIs transform the way that smoking cessation support is delivered in the future?
*We have added the following sentences to the implications for practice section to outline potential long-term implications of this research (p40): “In the long term, JITAIs have the potential to make smoking cessation support more dynamic, responsive, and adaptive to individual user needs. Future advancements in machine learning and passive sensing could further refine JITAIs' ability to predict relapse risk and deliver tailored interventions in real time. However, ethical considerations regarding data use, user autonomy, and accessibility will need to be continually reassessed to ensure these innovations do not exacerbate health inequalities. However, it is important to note that JITAIs need not and, by participants in this study, were not described as replacements for conventional face-to-face smoking cessation services but rather as additions that may make digital interventions more effective for those who choose to use them. As part of a comprehensive set of support options, JITAIs have the potential to complement existing services and provide additional, flexible assistance to those who may not otherwise engage with traditional stop-smoking programmes.”*

### Reviewer #3

Reviewer #3: About the paperwork
The paper titled "Exploring Perspectives on Digital Smoking Cessation Just-in-Time Adaptive Interventions: A Focus Group Study with Adult Smokers and Smoking Cessation Professionals" explores the views of adult smokers and smoking cessation professionals on just-in-time adaptive interventions (JITAIs) for smoking cessation. The study conducted focus groups with UK-based adult smokers and smoking cessation professionals between January and June 2024. The focus group discussions covered various topics, including the integration of JITAIs into users' lives, preferred content and features, and data privacy concerns.
Four major themes and 16 subordinate themes were identified through analysis:
1)Smoking Cessation Process: The non-linear and challenging journey of smoking cessation where JITAIs should provide consistent support.
2)JITAI characteristics: preferences for highly personalized interventions that offer both immediate support and in-depth content.
3)Perceived Value of the JITAI: The perceived usefulness and ease of use of a JITAI, with an emphasis on balancing convenience and disruption to facilitate behavior change.
4)Relationship with the JITAI: The desired supportive and non-judgmental relationship with a JITAI, promoting user autonomy.
In conclusion
The study highlights the need for JITAIs to be convenient, highly personalized, supportive, and non-judgmental while addressing privacy and data protection concerns. Smoking cessation professionals stressed the importance of privacy, while smokers had mixed opinions on this topic.
General comment
This paper provides valuable insights into the perspectives of both smokers and smoking cessation professionals on digital interventions for smoking cessation. The thorough analysis and identification of key themes offer a comprehensive understanding of user needs and preferences. The authors have successfully highlighted the importance of balancing convenience with effective disruption, personalizing interventions, and addressing privacy concerns. This study's findings have the potential to guide the development of more effective and user-friendly JITAIs, ultimately contributing to improved smoking cessation outcomes. Well done on such a well-structured and insightful research paper!

*We thank the reviewer for their kind comments!*
